# A prospective study of freezing of gait with early Parkinson disease in Chinese patients

**DOI:** 10.1097/MD.0000000000004056

**Published:** 2016-07-01

**Authors:** Hongbo Zhang, Xifan Yin, Zhiyuan Ouyang, Jing Chen, Shenghua Zhou, Changguo Zhang, Xin Pan, Shiliang Wang, Junxiang Yang, Yaoyao Feng, Ping Yu, Qiangchun Zhang

**Affiliations:** a Department of Neurology; b Department of Psychiatry, Third People's Hospital of Huzhou; c Department of Neurology, Second Affiliated Hospital, School of Medicine, Zhejiang University, Zhejiang Province, China.

**Keywords:** freezing of gait, Parkinson disease, risk factors

## Abstract

This study investigated the risk factors for freezing of gait (FOG) in the early stage of Parkinson disease in China, using a sample of 248 patients who were followed for 3 years. Part III of the Unified Parkinson Disease Rating Scale and the modified Hoehn-Yahr grading scale were used to evaluate the severity of motor symptoms. Nonmotor symptoms were assessed using the Hamilton Anxiety Rating Scale, Hamilton Depression Rating Scale (HAMD), and Non-Motor Symptoms Scale (NMSS). The end-point was the presence of FOG at the end of follow-up; patients with FOG were classified as freezers. The risk factors for FOG were analyzed at the end of the first, second, and third years after baseline. There were 40 freezers (16.13%) 1 year later, 98 (39.52%) 2 years later, and 128 (51.61%) 3 years later. FOG 3 years later was associated with the following variables: depression (*P* = 0.003), older age, living in the countryside, lower education, akinetic-rigid style, lower limbs as site of onset, early use of levodopa, higher daily dose of levodopa, and not using amantadine or selegiline and dopamine receptor agonists (*P* < 0.001). Early use of amantadine, selegiline, and dopamine receptor agonists was negatively related to FOG (*P* < 0.001). Binary logistic regression found that FOG was associated with lower education (odds ratio [OR] = 0.012, *P* < 0.001), akinetic-rigid style (OR = 4.881, *P* = 0.024), not using dopamine receptor agonists (OR = 4.324, *P* = 0.035), cognitive disturbances (OR = 0.331, *P* = 0.007), and sleep disorders (OR = 2.418, *P* = 0.036). However, the cardiovascular domain of the NMSS (OR = 2.729, *P* = 0.001) was the only risk factor for FOG 1 year later. Two years later, FOG was associated with mixed style (OR = 0.189, *P* = 0.005), lower limbs as site of onset (OR = 4.772, *P* = 0.008), not using dopamine receptor agonists (OR = 0.031, *P* < 0.001), and the anxiety/somatic domain of the HAMD (OR = 0.596, *P* = 0.033). Scores at baseline, patients with Parkinson disease were more likely to experience FOG if: they were older, or from the countryside; had an akinetic-rigid style, anxiety, or higher NMSS scores; they used levodopa early or did not use amantadine or selegiline; their lower limbs were the site of onset; or they had more severe motor disability or higher HAMD scores at baseline.

## Abstract 2

Supplemental Digital Content is available in the text

## 1 Introduction

Freezing of gait (FOG) is a common and disabling phenomena in Parkinson disease (PD) that usually is observed in its advanced stage: modified Hoehn–Yahr grading (H&Y) stage more than 2.5.^[1]^ It is a progressive manifestation of PD and it is one of the most common causes of falls.^[2]^ The definition of FOG is “a brief, episodic absence or a marked reduction of forward progression of the feet despite the intention to walk.”^[3]^ Patients report their feet seem to be suddenly glued to the floor as they try to initiate or maintain locomotion.^[4]^ FOG often leads to falls, injuries, secondary immobility, and reduced quality of life, thus, it has been investigated by a large number of studies.^[5–7]^

The pathophysiology of FOG is not well understood, although there are some hypotheses. First, FOG may emerge from changes in neuroanatomical networks in the brainstem, including the pedunculopontine nucleus and locus ceruleus, which are part of the mesencephalic locomotor center and postural control circuits.^[8]^ Second, an abnormality of the basal ganglia-brain stem loop may be a cause of FOG.^[9]^ Third, increasing evidence suggests that nonmotor systems are likely to be involved in its underlying mechanism.^[10]^ However, the relevant risk factors have not been identified. Several cross-sectional studies have analyzed the risk factors for FOG in advanced stages of PD.^[10,11]^ Nevertheless, there are few prospective studies of FOG in patients with early stage PD, even though such research would be very important for preventing FOG in PD. Therefore, exploring the risk factors for FOG has great clinical significance. Our study attempted to investigate the risk factors for FOG in the early stage of PD.

## 2 Methods

### 2.1 Participants

**Figure 1:**
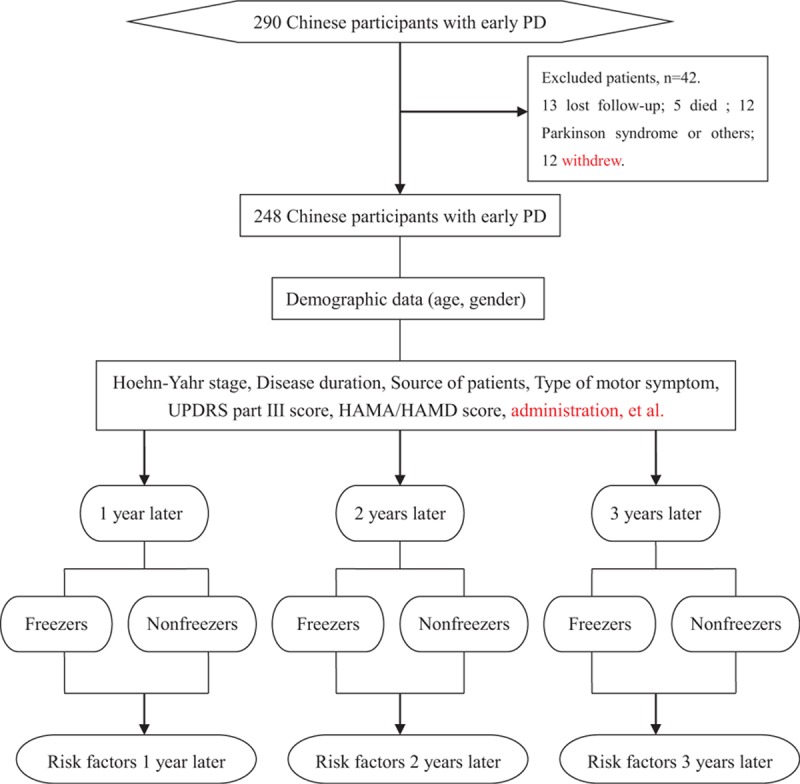
Study Roadmap. HAMA = Hamilton Anxiety Rating Scale, HAMD = Hamilton Depression Rating Scale, PD = Parkinson disease, UPDRS-III = Part III of Unified Parkinson Disease Rating Scale.

The study had got the approval of the ethics committee of the Third People's Hospital of Huzhou, Zhejiang Province, China. Patients were enrolled in the study consecutively. All participants were recruited through the third People's Hospital of Huzhou, Zhejiang Province of China. The study roadmap is shown in Fig. 1. All participants were diagnosed according to the Unified Kingdom PD Society Brain Bank Clinical Diagnostic Criteria for PD^[12]^ and they had never taken anti-Parkinson drugs. Patients with atypical and secondary Parkinsonism were excluded from the study. All patients were in the early-stages of PD and never had FOG. They were regularly treated according to Chinese guidelines for the treatment of PD after receiving their diagnosis.^[13]^ Patients with anxiety or depression were diagnosed by at least one of trained psychiatrists, in addition to completing the Hamilton Anxiety Rating Scale (HAMA) and Hamilton Depression Rating Scale (HAMD). Patients with depression were treated regularly with antidepressants.

Table 1 shows the demographic characteristics and clinical details of the participants at baseline. All clinical items were assessed by an experienced neurologist. The clinical data included disease duration, type of motor symptoms, site of initial motor symptoms, H&Y stage, and scores on Part III of Unified Parkinson Disease Rating Scale (UPDRS-III), the HAMD, and the HAMA. Early-onset PD (EOPD) was defined as onset at an age younger than 50 years old, whereas late-onset PD (LOPD) was defined as onset at 50 years of age or older. Patients were grouped into 3 subtypes, including tremor-dominant, akinetic-rigid, and mixed, according to the criteria described in a previous study.^[14]^ The UPDRS-III and H&Y stage were used to evaluate the severity of the motor symptoms. The degree of depression, anxiety, and nonmotor symptoms were assessed using the HAMA, the HAMD, and the Non-Motor Symptoms Scale (NMSS), respectively.

**Table 1:**
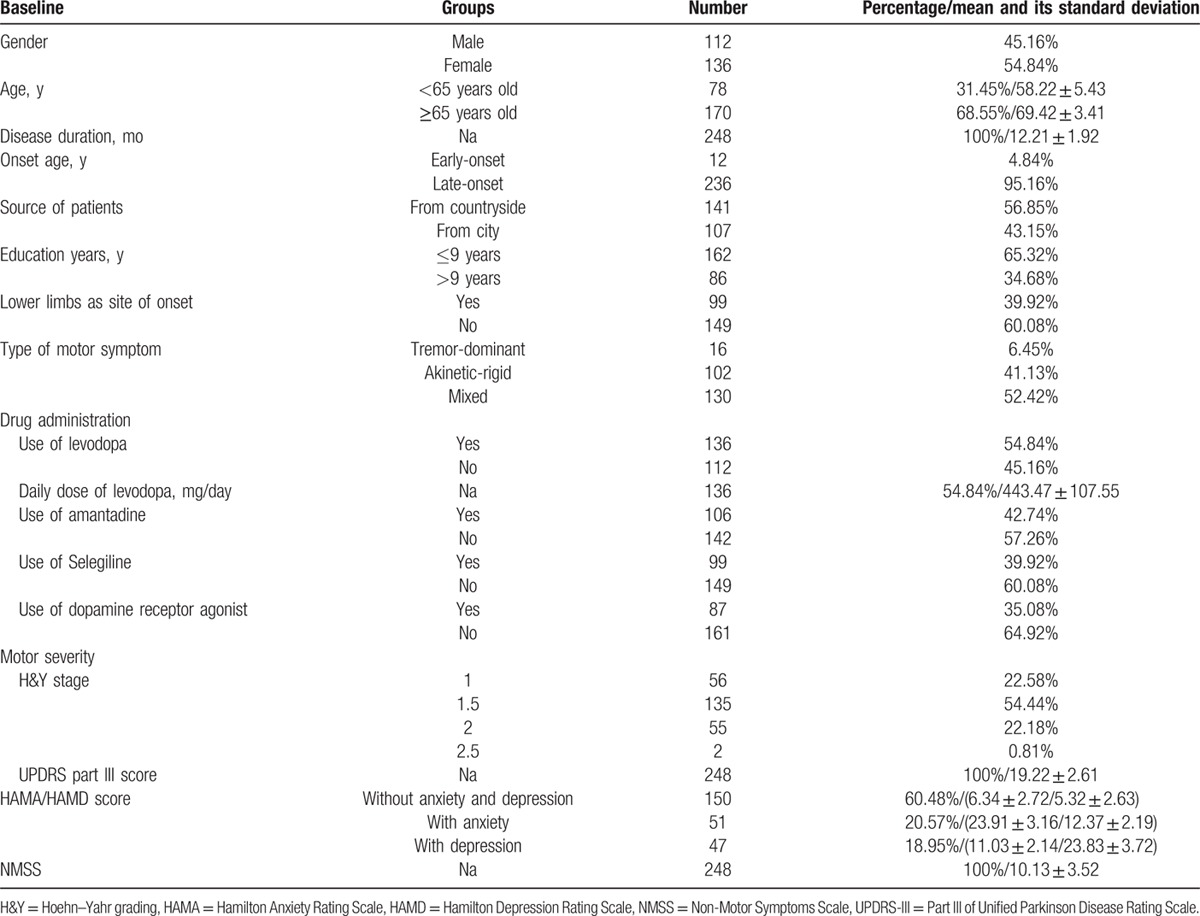
The demographic characteristics and clinical details of 248 participants at baseline.

### 2.2 Assessment of FOG

FOG was identified by 2 criteria used in a previous study:^[15]^ (i) a convincing subjective report of FOG that was consistent with the characteristics of the phenomenon, especially the typical feeling that the feet were glued to the floor; and (ii) a patient's recognition of the typical FOG phenomenon when it was demonstrated to him or her by an experienced clinician.^[16]^ We mainly determined FOG by patients’ responses to the question: “Do you feel that your feet get glued to the floor while walking, making a turn, or when trying to initiate walking?” Patients who answered “yes” were identified as freezers. FOG was also identified when it was observed by an experienced neurologist during a visit, or it was reported by the patients, their family members, or their caregivers when it occurred at home or anywhere outside of the hospital.

### 2.3 Procedure

The patients were followed for 3 years. The first patient was enrolled on March 2, 2010 and the last patient was enrolled on September 4, 2012. We completed the 3-year follow-up on September 4, 2015. We evaluated the patients at baseline, 1 year later, 2 years later, and 3 years later. The data collected at baseline included age, gender, onset age, disease duration, type of motor symptoms, site of initial motor symptoms, H&Y stage, scores on the HAMA, HAMD, and UPDRS-III, and other information shown in Table 1. We observed changes in prescriptions and mood status at the 3-year follow-up (Table 6). The patients were divided into freezers and non-freezers based on the presence of FOG.

**Table 6:**
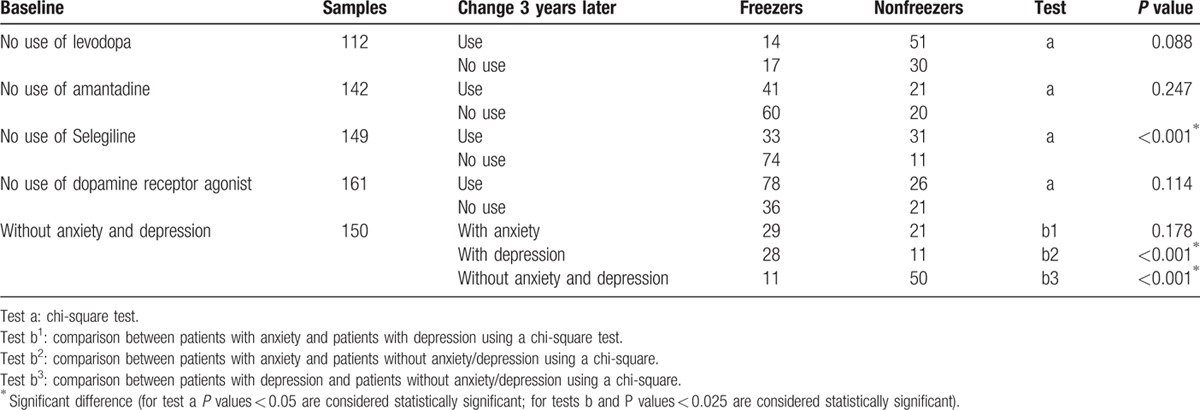
Associations between FOG 3 years later and changes in drug administration and mood.

### 2.4 Statistical analysis

All analyses were performed using the Statistical Package for the Social Sciences version 19.0 for Windows. All continuous data, such age, disease duration, daily levodopa dose, and HAMA, HAMD, and UPDRS-III scores are presented as the mean ± the standard deviation. Student *t*-tests were used to compare continuous variables between patients with and without FOG. The chi-square test was used to evaluate differences in categorical variables between patients with and without FOG. Analysis of covariance, adjusting for age, was performed to compare the total scores and the domain scores for the HAMD, HAMA, and NMSS between patients with and without FOG. A binary logistic regression model was used to explore potential factors related to FOG. The presence or absence of FOG was used as the dependent variable, and independent variables included: age, source of patients, years of education, lower limbs as the site of onset, type of motor symptom, drug administration, the NMSS scores for the cardiovascular, sleep/fatigue, mood/apathy, perceptual problems/hallucinations, attention/memory, gastrointestinal, and urinary domains as well as the HAMD domain scores for anxiety/somatic, weight, cognitive disturbance, block, sleep disorder, and feelings of despair. All statistical tests were 2-tailed, and *P* < 0.05 was considered to be statistically significant (for multiple comparisons of the chi-square test, *P* < 0.025 was considered to be statistically significant).

## 3 Results

### 3.1 Participants’ demographic characteristics and clinical details

A total of 290 Chinese patients with early PD (H&Y stage ≤ 2.5) were consecutively recruited to examine changes in the prevalence of FOG and its related risk factors through a 3-year follow-up. There were 13 patients lost in the follow-up, 5 patients died, and 12 patients eventually proved to have Parkinson syndrome or other nervous system degeneration diseases, and 12 patients withdrew because of serious fractures, cardiovascular events, or stoke. Finally, 248 participants with early PD completed the 3-year follow-up and were included in the analyses. Of the 248 patients, 112 cases were male (45.16%) and 136 cases were female (54.84%). There were 78 patients who were less than 65 years old (31.45%) and 170 patients who were more than or equal to 65 years old (68.55%). The EOPD and LOPD patients accounted for 4.84% and 95.16% of the sample, respectively. Table 1 shows the details of the variables.

### 3.2 Association between variables at baseline and FOG after 3 years

Forty PD patients (16.13%) reported FOG one year later (ie, 1 year after baseline), 98 (39.52%) reported FOG after 2 years, and 128 (51.61%) reported FOG 3 years later. Table 2 shows the relationship between FOG 3 years later and related variables at baseline. There was no significant difference in the prevalence of FOG between male and female patients (45.16% vs 54.84%, *P* = 0.474), or the EOPD and LOPD patients (4.84% vs 95.16%, *P* = 0.059), and there was no difference in UPDRS-III scores between freezers and nonfreezers (20.00 ± 2.47 vs 18.37 ± 2.51, *P* = 0.905).

**Table 2:**
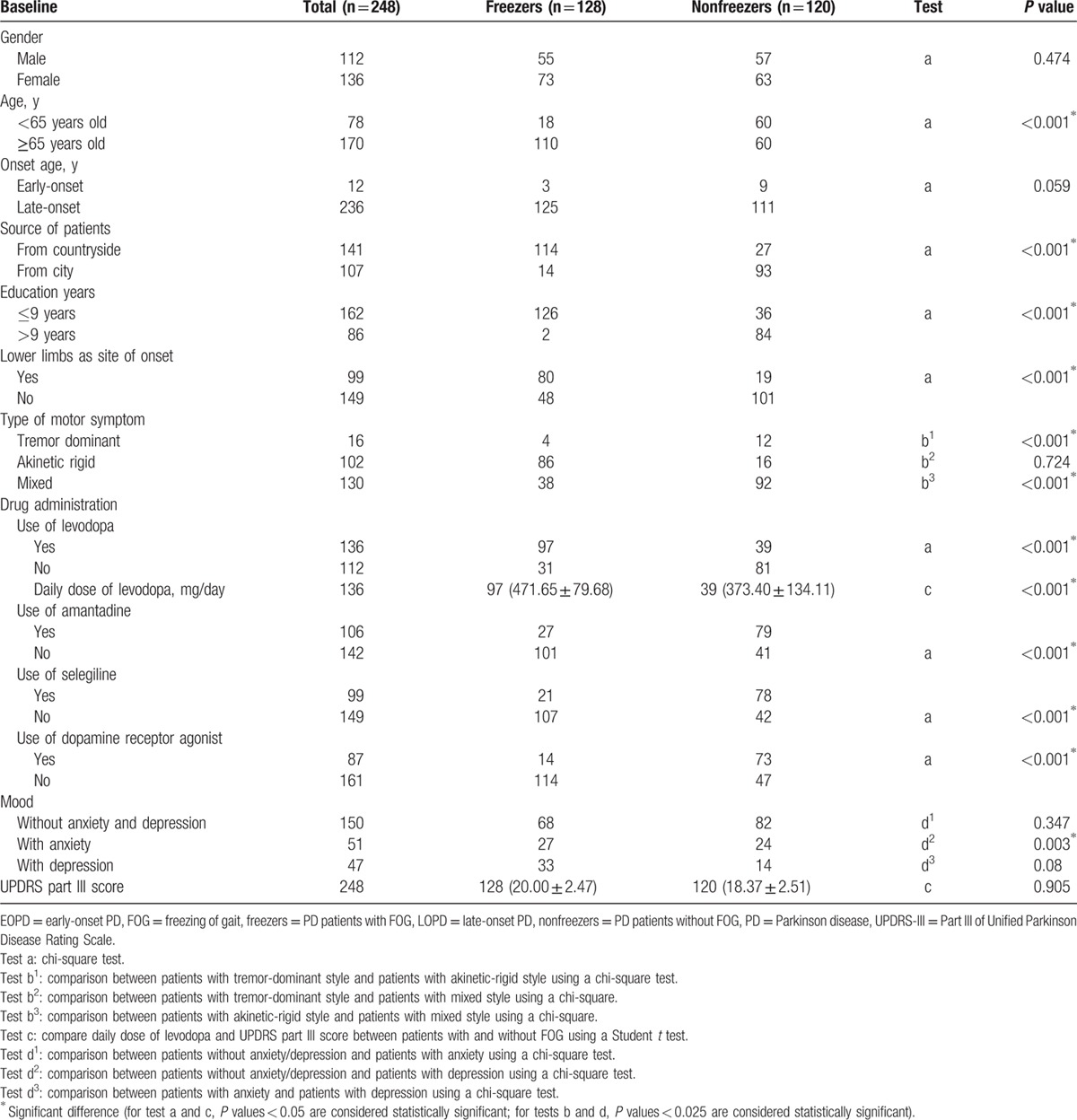
Association between variables at baseline and FOG after 3 years.

FOG occurred more frequently in patients older than 65 years old than in patients younger than 65 years old (68.55% vs 31.45%, *P* < 0.001). Patients who were from the countryside (56.85% vs 43.15%, *P* < 0.001), or had a lower education were more prone to have FOG (65.32% vs 34.68%, *P* < 0.001). Patients whose lower limbs were the site of onset were more likely to develop FOG than those whose upper limbs were the site of onset (39.92% vs 60.08%, *P* < 0.001). Patients with an akinetic-rigid style also were more likely to suffer from FOG (*P* < 0.001). With respect to drug use, early use of levodopa seemed to increase FOG (54.84% vs 45.16%, *P* < 0.001). Patients with a higher daily dose of levodopa had a higher incidence of FOG (*P* < 0.001), whereas early use of amantadine (42.74% vs 57.26%, *P* < 0.001), selegiline (39.92% vs 60.08%, *P* < 0.001), and dopamine receptor agonists (35.08% vs 64.92%, *P* < 0.001) appeared to reduce FOG. Patients with depression had a higher rate of FOG (*P* = 0.003).

Table 3 shows the relationship between NMSS scores at baseline and FOG 3 years later. After adjusting for age, patients with FOG (freezers) had a significantly higher NMSS total score and higher scores on the cardiovascular, sleep/fatigue, mood/apathy, perceptual problems/hallucinations, attention/memory, gastrointestinal, and urinary domains of the NMSS compared with nonfreezers (*P* < 0.05). However, there were no differences in the scores for the sexual dysfunction or miscellaneous domains of the NMSS between the freezers and nonfreezers (*P* > 0.05).

**Table 3:**
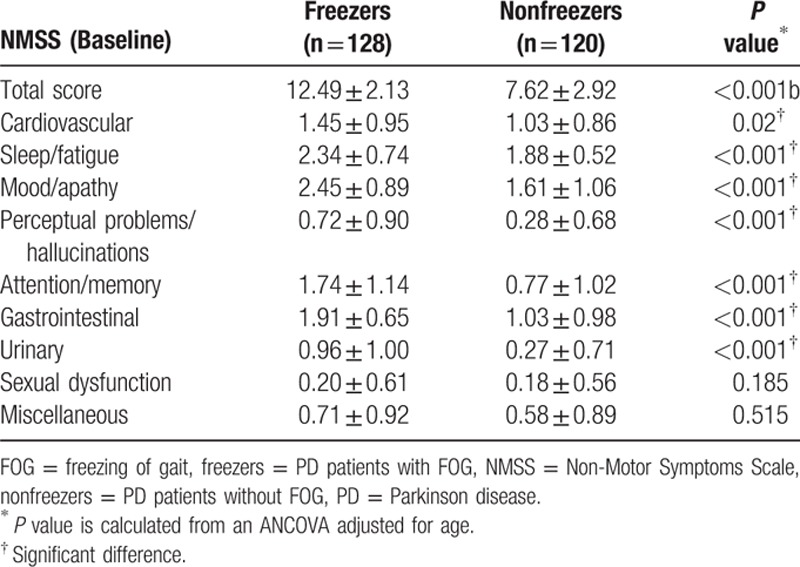
Associations between factors in NMSS at baseline and FOG 3 years later in PD patients.

Table 4 shows the relationship between HAMA&HAMD scores at baseline and FOG. After adjusting for age, freezers had higher total HAMD scores as well as higher scores on the anxiety/somatic, weight, cognitive disturbance, block, sleep disorder, and feelings of despair domains of the HAMD compared with nonfreezers (*P* < 0.05). However, there was no group difference in the scores for the somatic anxiety or the psychic anxiety domains of the HAMA or the diurnal variations domain of the HAMD (*P* > 0.05).

**Table 4:**
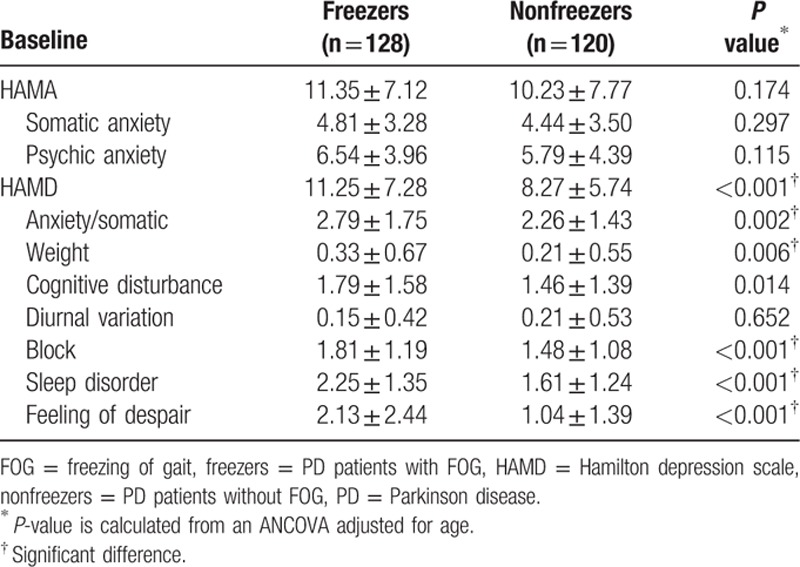
Associations between HAMA/HAMD scores at baseline and FOG 3 years later in PD patients.

The baseline variables potentially related to FOG 3 years later are presented in Table 5. The results indicated that lower education, akinetic-rigid style, no use of dopamine receptor agonists, higher scores for cognitive disturbances, and higher scores for sleep disorders were associated with FOG (*P* < 0.05). No significant associations were found between the remaining variables and FOG.

**Table 5:**
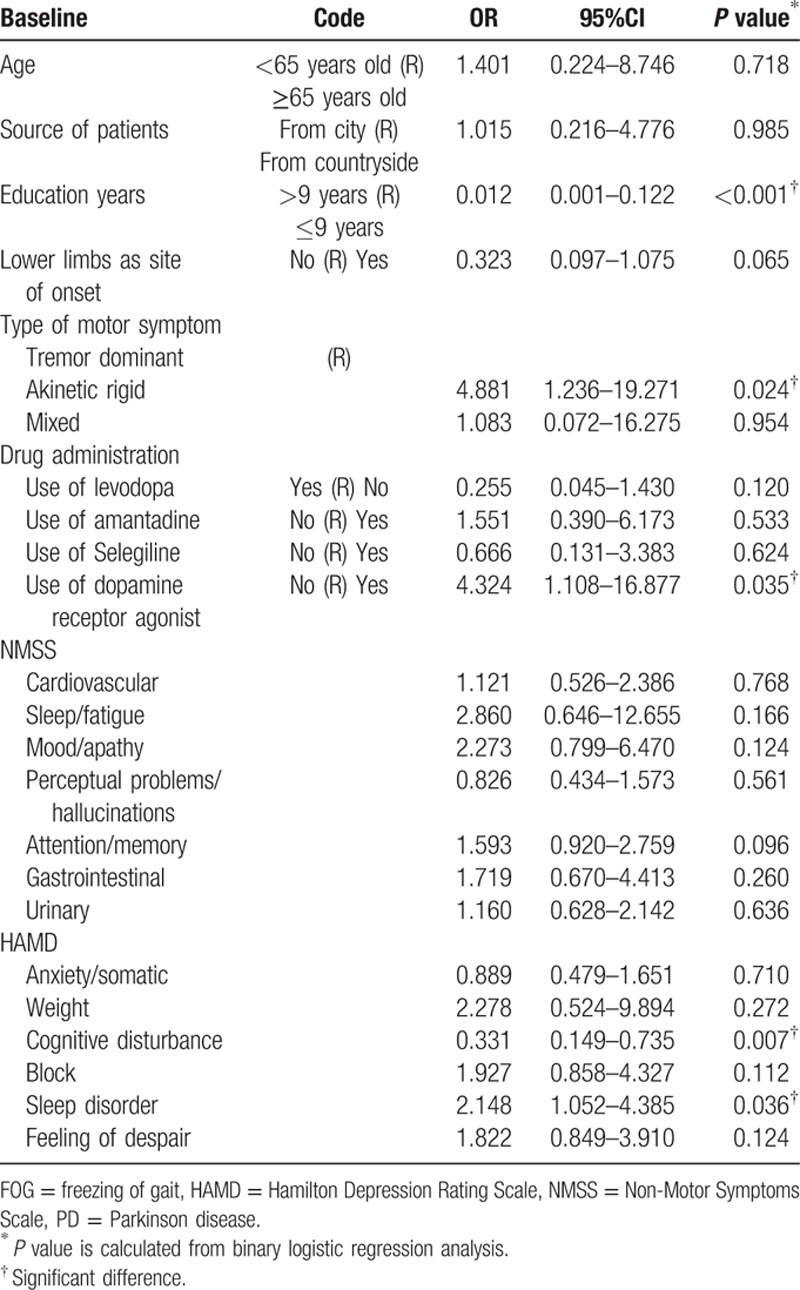
Associations between clinical factors at baseline and FOG 3 years later in PD patients.

### 3.3 Relationships between the changes in drug use, mood, and FOG

We analyzed the association of FOG: (a) with changes between the baseline and follow-up use of levodopa, amantadine, selegiline, and dopamine receptor agonists; and (b) changes in patient anxiety and depression between baseline and follow-up. The chi-square test was used to compare the difference between freezers and nonfreezers 3 years after baseline (Table 6). The analyses found no differences between use and no use of levodopa, amantadine, and dopamine receptor agonists (*P* > 0.05). In contrast, administering selegiline during the course of the disease appeared to reduce FOG (*P* < 0.001). Patients who developed anxiety or depression during the course of disease were more likely to develop FOG (*P* < 0.001).

### 3.4 Association between factors at baseline and FOG after 1 and 2 years

We analyzed the association between FOG 1 year after baseline and potential predictor variables 3 years after baseline using binary logistic regression (the same method was used for 2 years after baseline). Table 7 shows that a higher score for the cardiovascular domain on the NMSS was significantly associated (*P* = 0.001) with FOG 1 year later (i.e., 1 year after baseline). The lower limbs as the site of onset (*P* = 0.008), mixed style (*P* = 0.005), no use of dopamine receptor agonists (*P* < 0.001), and a higher score on the anxiety/somatic domain of the HAMD (*P* = 0.033) were significantly associated with FOG 2 years later (ie, 2 years after baseline).

**Table 7:**
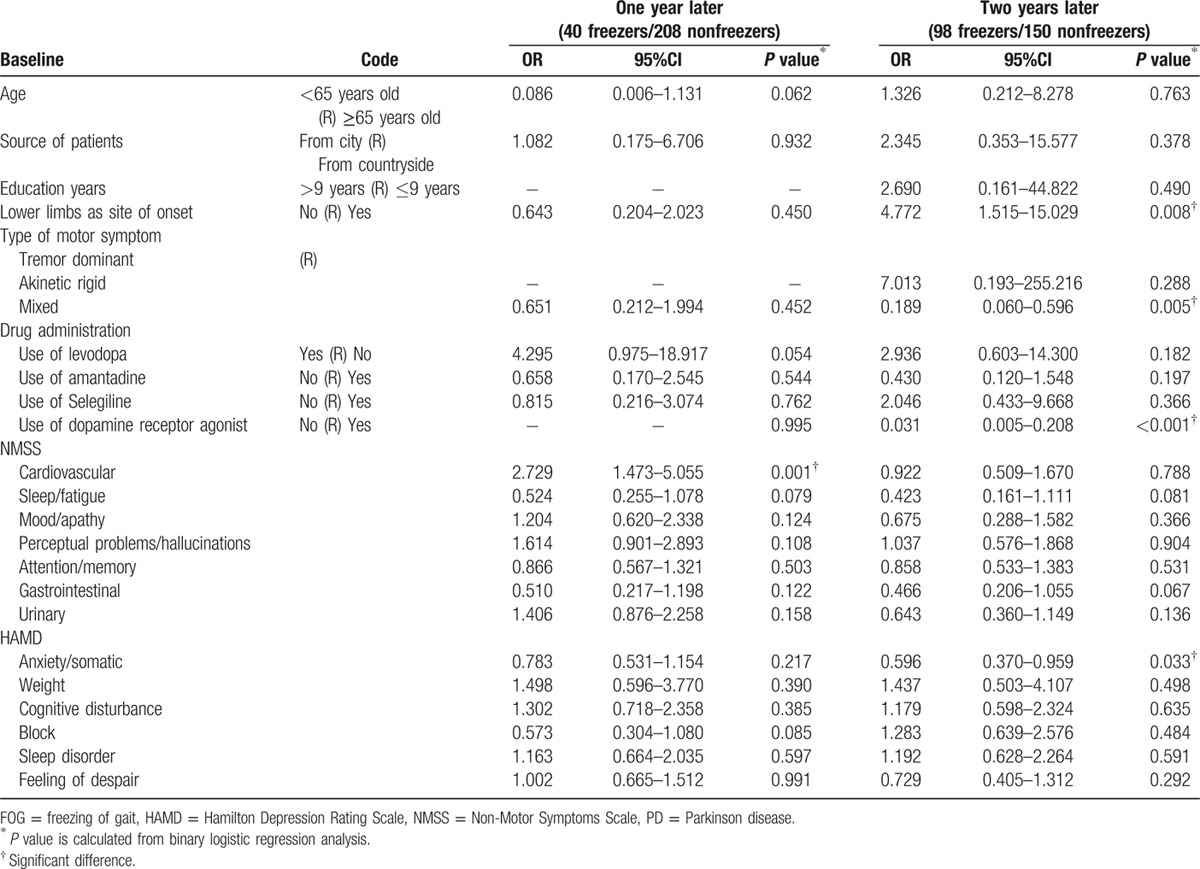
Associations between risk factors at baseline and FOG one or 2 years later in PD patients.

## 4 Discussion

PD is a common disease. When FOG occurs early in PD, it always has a mild form, and early detection of FOG is an alarm signal that may cast doubt on a diagnosis of PD.^[17]^ The prevalence of FOG is higher in older patients, many of whom have osteoporosis and other multisystem diseases. FOG is one of the major disabling symptoms of advanced PD, as severe FOG can lead to falls, pain, skin contusions, fractures, and activity limitations.^[18–20]^ Hence, early identification, treatment, and control of risk factors for FOG are very important. To our knowledge, this is the first prospective study to investigate the prevalence and clinical correlates of FOG in Chinese patients with early PD.

We found FOG was very common in the Chinese population of PD patients; it was 16.13% one year later—that is, 1 year after baseline—39.52% 2 years later, and 51.61% 3 years later. These findings are consistent with previous studies of non-Asian populations (prevalence ranging from 32% to 72%).^[21–26]^ The year-by-year increase in the incidence of FOG suggests that older patients or patients with longer durations of PD are more likely to experience FOG.

We found that patients who had a lower education or were from the countryside were more likely to suffer from FOG. This could be because patients with a higher education had a better understanding of PD and had better compliance, whereas patients from cities were closer to hospitals where they could be treated. Additionally, the results showed that patients whose lower limbs were the site of onset and patients with an akinetic-rigid style were more likely to suffer from FOG. Several studies have reported that patients with onset in the lower limbs were prone to experience FOG.^[11,27,28]^ Our study suggests that early use of levodopa and a higher daily dose of levodopa at baseline were associated with a higher incidence of FOG, which is in accordance with studies by Ou and Macht.^[11,25]^ Furthermore, the result indicated that early use of amantadine, selegiline, and dopamine receptor agonists can reduce FOG, which is consistent with some previous studies.^[11,25,29,30]^ The results also showed that patients with depression were more likely to suffer from FOG, which is consistent with a previous study that found patients with anxiety or depression were more likely to experience FOG.^[10]^ Our study found no difference in FOG between male and female patients, which is consistent with a previous study on English patients.^[26]^ Nor was there any difference in FOG between early-onset and late-onset patients. This could be due to the fact that there were very few early-onset patients enrolled in the study, which might have introduced a statistical bias. Furthermore, patients who had both anxiety and depression at baseline or later in the study were more likely to suffer from FOG than patients who did not have these combined affective disorders. Giladi study suggested that the use of selegiline later in PD could reduce FOG,^[31]^ and our study found similar results, in addition to the finding that other drugs did not reduce FOG.

**Figure 2:**
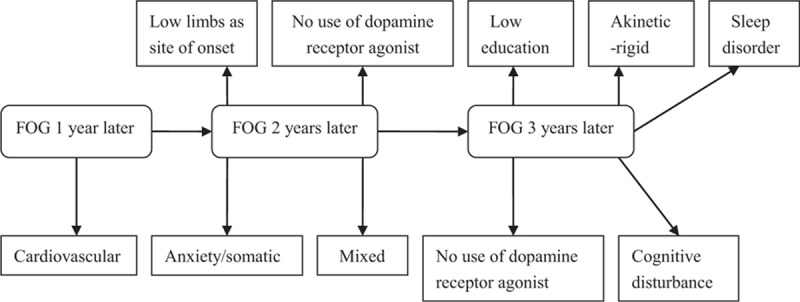
FOG and its risk factors. FOG = freezing of gait.

We analyzed the association between the risk factors at baseline and FOG 3 years later using a binary logistic regression model. The model only adjusted for age due to the uniformity of early PD patients and the very similar course of disease. The results indicated that FOG was associated with lower education, an akinetic-rigid style, not using dopamine receptor agonists, and higher scores on the cognitive disturbance domain and higher scores on the sleep disorder domain of the HAMD. The longer observation time, the higher the accuracy of the association between risk factors and FOG. Therefore, we also used binary logistic regression to determine whether the risk factors 3 years later were the same risk factors observed at 1 year and 2 years later. The results showed that a higher score on the cardiovascular domain of the NMSS at baseline was associated with FOG 1 year later (Fig. 2). Lower limb site of onset, mixed style, not using dopamine receptor agonists, and a higher score on the anxiety/somatic domain of the HAMD were associated with FOG 2 years later (Fig. 2). Though the risk factors changed year by year, we could see that nonmotor symptoms, in addition to movement disorders, had an important role in the occurrence of FOG. Thus, higher scores on the NMSS or HAMA and HAMD in the early stage of PD predicted the early occurrence of FOG. Not using dopamine receptor agonists was a higher risk factor for FOG, which means that early use of dopamine receptor agonists could benefit early PD patients.

Many patients in our study reported falls, skin contusions, and fractures because of severe FOG. Thus, physicians should pay attention not only to the adjustment of drugs, but also to the prevention of FOG. The key way to prevent FOG is to address non-motor symptoms in early PD patients. Doctors should instruct patients and their family members, especially those who have a lower education or live in the countryside, to improve patients’ compliance with treatment. Further research is needed to explore the degree to which controlling for some of the controllable risk factors of FOG can delay the occurrence of FOG or reduce FOG.

### 4.1 Limitations

There were some limitations of our study that should be discussed. First, FOG occurs more commonly at home, which makes it difficult to evaluate by physicians or researchers. Therefore, recall bias might have influenced the results of the study. Second, the results might have been affected by some unpredictable factors, such as the progress of the disease. Third, the drug compliance of each patient was different, and the anti-Parkinson and antianxiety drugs that were used also were different.

## 5 Conclusions

FOG is a common disabling symptom of PD in Chinese patients. Risk factors for FOG in patients with early PD included: older age, being from the countryside, having an akinetic-rigid style, having anxiety or depression, early use of levodopa, not using amantadine or selegiline and dopamine receptor agonists, the lower limbs as the site of onset, more severe motor disability, and higher scores on the HAMD or NMSS at baseline. Lower education, not using dopamine receptor agonists at baseline, an akinetic-rigid style, and especially, cognitive disturbances and sleep disorders (as measured by the HAMD) are strongly associated with FOG.

## Supplementary Material

Supplemental Digital Content
