# Supplementary material for: A prospective study of freezing of gait with early Parkinson disease in Chinese patients
Source: Medicine (Baltimore). 2016 Jul 1;95(26):e4056. doi: 10.1097/MD.0000000000004056 (PMC4937955; doi:10.1097/MD.0000000000004056)
Supplement: Supplemental Digital Content [file medi-95-e4056-s001.pdf]

A prospective study of freezing of gait in Chinese patients with early Parkinson's disease

Hongbo Zhang[1a*], MD; Xifan Yin[1b**], MD; Zhiyuan Ouyang[2*], MD; Jing Chen[1a], MD; Shenghua Zhou[1a], MD; Changguo Zhang[1a], MD; Xin Pan [1b], MD; Shiliang Wang [1b], MD; ,Junxiang Yang[1a], MD; Yaoyao Feng[1a], MD, Ping Yu[1a], MD

1a Department of Neurology, Third People's Hospital of Huzhou, Zhejiang Province, China

1b Department of Psychiatry, Third People's Hospital of Huzhou, Zhejiang Province, China

2 Department of Neurology, Second Affiliated Hospital, School of Medicine, Zhejiang University, Zhejiang Province, China

* These authors contributed equally to this work and share first authorship.

** Corresponding author. Tel: +8615268245819, E-Mail: xifan82@sina.com

All authors certify that our manuscript is a unique submission and is not being considered for publication by any other source in any medium. Further, the manuscript has not been published, in part or in full, in any form.

Supplementary Table S1. The severity of the motor symptoms of 248 patients was evaluated by modified Hoehn and Yahr Staging and Part III of Unified Parkinson Disease Rating Scale (UPDRS-III) at baseline.

modified Hoehn and Yahr Staging/ scores of UPDRS-III	Number	Percentages/ scores
Stage 0	0	0%
Stage 1	56	22.58%
Stage 1.5	135	54.44%
Stage 2	55	22.18%
Stage 2.5	2	0.81%
Stage 3	0	0%
Stage 4	0	0%
Stage 5	0	0%
scores of UPDRS-III	248	19.22±2.61

MODIFIED HOEHN AND YAHR STAGING
Stage 0 —No signs of disease.
Stage 1 —Unilateral disease.
Stage 1.5 —Unilateral plus axial involvement.
Stage 2 —Bilateral disease, without impairment of balance.
Stage 2.5 —Mild bilateral disease with recovery on pull test.
Stage 3 —Mild to moderate bilateral disease; some postural instability; physically independent.
Stage 4 —Severe disability; still able to walk or stand unassisted.
Stage 5 —Wheelchair bound or bedridden unless aided.

Part III of Unified Parkinson Disease Rating Scale
18. Speech
0 = Normal.
1 = Slight loss of expression, diction and/or volume.
2 = Monotone, slurred but understandable; moderately impaired.
3 = Marked impairment, difficult to understand.
4 = Unintelligible.
19. Facial Expression
0 = Normal.
1 = Minimal hypomimia, could be normal "Poker Face".
2 = Slight but definitely abnormal diminution of facial expression.
3 = Moderate hypomimia; lips parted some of the time.
4 = Masked or fixed facies with severe or complete loss of facial expression; lips parted 1/4 inch or more.
20. Tremor at rest (head, upper and lower extremities)
0 = Absent.
1 = Slight and infrequently present.
2 = Mild in amplitude and persistent. Or moderate in amplitude, but only intermittently present.

3 = Moderate in amplitude and present most of the time.
4 = Marked in amplitude and present most of the time.
21. Action or Postural Tremor of hands
0 = Absent.
1 = Slight; present with action.
2 = Moderate in amplitude, present with action.
3 = Moderate in amplitude with posture holding as well as action.
4 = Marked in amplitude; interferes with feeding.
22. Rigidity (Judged on passive movement of major joints with patient relaxed in sitting position. Cogwheeling to be ignored.)
0 = Absent.
1 = Slight or detectable only when activated by mirror or other movements.
2 = Mild to moderate.
3 = Marked, but full range of motion easily achieved.
4 = Severe, range of motion achieved with difficulty.
23. Finger Taps (Patient taps thumb with index finger in rapid succession.)
0 = Normal.
1 = Mild slowing and/or reduction in amplitude.
2 = Moderately impaired. Definite and early fatiguing. May have occasional arrests in movement.
3 = Severely impaired. Frequent hesitation in initiating movements or arrests in ongoing movement.
4 = Can barely perform the task.
24. Hand Movements (Patient opens and closes hands in rapid succesion.)
0 = Normal.
1 = Mild slowing and/or reduction in amplitude.
2 = Moderately impaired. Definite and early fatiguing. May have occasional arrests in movement.
3 = Severely impaired. Frequent hesitation in initiating movements or arrests in ongoing movement.
4 = Can barely perform the task.
25. Rapid Alternating Movements of Hands (Pronation-supination movements of hands, vertically and
horizontally, with as large an amplitude as possible, both hands simultaneously.)
0 = Normal.
1 = Mild slowing and/or reduction in amplitude.
2 = Moderately impaired. Definite and early fatiguing. May have occasional arrests in movement.
3 = Severely impaired. Frequent hesitation in initiating movements or arrests in ongoing movement.
4 = Can barely perform the task.
26. Leg Agility (Patient taps heel on the ground in rapid succession picking up entire leg. Amplitude
should be at least 3 inches.)
0 = Normal.
1 = Mild slowing and/or reduction in amplitude.
2 = Moderately impaired. Definite and early fatiguing. May have occasional arrests in movement.

3 = Severely impaired. Frequent hesitation in initiating movements or arrests in ongoing movement.
4 = Can barely perform the task.
27. Arising from Chair
(Patient attempts to rise from a straightbacked chair, with arms folded across chest.)
0 = Normal.
1 = Slow; or may need more than one attempt.
2 = Pushes self up from arms of seat.
3 = Tends to fall back and may have to try more than one time, but can get up without help.
4 = Unable to arise without help.
28. Posture
0 = Normal erect.
1 = Not quite erect, slightly stooped posture; could be normal for older person.
2 = Moderately stooped posture, definitely abnormal; can be slightly leaning to one side.
3 = Severely stooped posture with kyphosis; can be moderately leaning to one side.
4 = Marked flexion with extreme abnormality of posture.
29. Gait
0 = Normal.
1 = Walks slowly, may shuffle with short steps, but no festination (hastening steps) or propulsion.
2 = Walks with difficulty, but requires little or no assistance; may have some festination, short steps,
or propulsion.
3 = Severe disturbance of gait, requiring assistance.
4 = Cannot walk at all, even with assistance.
30. Postural Stability (Response to sudden, strong posterior displacement produced by pull on shoulders
while patient erect with eyes open and feet slightly apart. Patient is prepared.)
0 = Normal.
1 = Retropulsion, but recovers unaided.
2 = Absence of postural response; would fall if not caught by examiner.
3 = Very unstable, tends to lose balance spontaneously.
4 = Unable to stand without assistance.
31. Body Bradykinesia and Hypokinesia (Combining slowness, hesitancy, decreased armswing, small
amplitude, and poverty of movement in general.)
0 = None.
1 = Minimal slowness, giving movement a deliberate character; could be normal for some persons.
Possibly reduced amplitude.
2 = Mild degree of slowness and poverty of movement which is definitely abnormal.
Alternatively, some reduced amplitude.
3 = Moderate slowness, poverty or small amplitude of movement.
4 = Marked slowness, poverty or small amplitude of movement.

Supplementary Table S2. Non-motor symptoms were assessed using the Hamilton Anxiety Rating Scale (HAMA), Hamilton Depression Rating Scale (HAMD), and Non-Motor Symptoms Scale (NMSS) at baseline.

	Nember/ Percentages	Scores
HAHA/HAMD Scores	248/100%	
Without anxiety and depression	150/60.48%	6.34±2.72/5.32±2.63
With anxiety	51/20.57%	23.91±3.16/12.37±2.19
With depression	47/18.95%	11.03±2.14/23.83±3.72
NMSS scores	248/100%	10.13±3.52

The Hamilton Anxiety Scale (HAM-A) is a rating scale developed to quantify the severity of anxiety symptomatology,often used in psychotropic drug evaluation. It consists of 14 items, each defined by a series of symptoms. Each item is rated on a 5-point scale, ranging from 0 (not present) to 4 (severe).

0 = Not present to 4 = Severe

1. ANXIOUS MOOD
• Worries
• Anticipates worst
2. TENSION
• Startles
• Cries easily
• Restless
• Trembling
3. FEARS
• Fear of the dark
• Fear of strangers
• Fear of being alone
• Fear of animal
4. INSOMNIA
• Difficulty falling asleep or staying asleep
• Difficulty with Nightmares
5. INTELLECTUAL
• Poor concentration
• Memory Impairment
6. DEPRESSED MOOD
• Decreased interest in activities
• Anhedoni
• Insomnia
7. SOMATIC COMPLAINTS: MUSCULAR
• Muscle aches or pains
• Bruxism
8. SOMATIC COMPLAINTS: SENSORY
• Tinnitus

• Blurred vision
9. CARDIOVASCULAR SYMPTOMS
• Tachycardia
• Palpitations
• Chest Pain
• Sensation of feeling faint
10. RESPIRATORY SYMPTOMS
• Chest pressure
• Choking sensation
• Shortness of Breath
11. GASTROINTESTINAL SYMPTOMS
• Dysphagia
• Nausea or Vomiting
• Constipation
• Weight loss
• Abdominal fullness
12. GENITOURINARY SYMPTOMS
• Urinary frequency or urgency
• Dysmenorrhea
• Impotence
13. AUTONOMIC SYMPTOMS
• Dry Mouth
• Flushing
• Pallor
• Sweating
14. BEHAVIOR AT INTERVIEW
• Fidgets
• Tremor
• Paces

The Hamilton Depression Scale was NOT designed to be self-administered because of the the difficulty of interpretation of some of the clinical terms. This online version is simply a tool to facilitate scoring by an experience psychiatrist or other health care professional with knowledge of psychiatry.

For each item check the description that best characterises the patient during the past week

After answering ALL of the items click on the 'Score' button. A new window will open with the score and interpretation.

1. DEPRESSED MOOD
(Sadness, hopeless, helpless, worthless)

Absent

These feelings are indicated only on questioning

These feelings are spontaneously reported verbally

- [ ] Communicates feelings non-verbally i.e., through facial expression, posture, voice, and tendency to weep
- [ ] Patient reports VIRTUALLY ONLY these feelings in his spontaneous verbal and non-verbal communication

2. FEELINGS OF GUILT

- [x] Absent
- [ ] Self reproach, feels he has let people down
- [ ] Ideas of guilt or rumination over past errors or sinful deed
- [ ] Present illness is a punishmnent. Delusions of guilt
- [ ] Hears accusatory or denunciatory voices and/or experiences threatening visual hallucinations

3. SUICIDE

- [x] Absent
- [ ] Feels life is not worth living
- [ ] Wishes he were dead or any thoughts of possible death to self
- [ ] Suicide ideas or gesture
- [ ] Attempts at suicide (any serious attempt rates)

4. INSOMNIA EARLY

- [x] No difficulty falling asleep
- [ ] Complains of occasional difficulty falling asleep - more than 1/2 hour
- [ ] Complains of nightly difficulty falling asleep

5. INSOMNIA MIDDLE

- [x] No difficulty
- [ ] Patient complains of being restless and disturbed during the night
- [ ] Waking during the night - any getting out of bed (except for purposes of voiding)

6. INSOMNIA LATE

- [x] No difficulty

- Waking in early hours of the morning but goes back to sleep
- Unable to fall asleep again if he gets out of bed

7. WORK AND ACTIVITIES

- No difficulty
- Thoughts and feelings of incapacity, fatigue or weakness related to activities (work or hobbies)
- Loss of interest in activities (hobbies or work) - either directly reported by patient, or indirectly in listlessness, indecision and vacillation (feels he has to push himself to work or do activities)
- Decrease in actual time spent in activities or decrease in productivity. In hospital, if patient does not spend at least three hours a day in activities (hospital job or hobbies) exclusive of ward chores
- Stopped working because of present illness. In hospital, if patient engages in no activities except ward chores, or if patient fails to perform ward chores unassisted

8. RETARDATION: PSYCHOMOTOR
(Slowness of thought and speech; impaired ability to concentrate; decreased motor activity)

- Normal speech and thought
- Slight retardation at interview
- Obvious retardation at interview
- Interview difficult
- Complete stupor

9. AGITATION

- None
- Fidgetiness
- Playing with hands, hair,etc
- Moving about, can't sit still
- Hand wringing, nail biting, hair-pulling, biting of lips

10. ANXIETY: PSYCHIC

- [x] No difficulty
- [ ] Subjective tension and irritability
- [ ] Worrying about minor matters
- [ ] Apprehensive attitude apparent in face or speech
- [ ] Fears expressed without questioning

11. ANXIETY: SOMATIC
(Physiological concomitants of anxiety, such as - Gastro-intestinal: dry mouth, wind, indigestion, diarrhea, cramps, belching. - Cardio-vascular : palpitations, headaches. - Respiratory: hyperventilation, sighing. - Urinary frequency - Sweating)

- [x] Absent
- [ ] Mild
- [ ] Moderate
- [ ] Severe
- [ ] Incapacitating

12. SOMATIC SYMPTOMS: GASTROINTESTINAL

- [x] None
- [ ] Loss of appetite but eating without staff encouragement. Heavy feelings in abdomen
- [ ] Difficulty eating without staff urging. Requests or requires laxatives or medication for bowels or medication for gastro-intestinal symptoms

13. SOMATIC SYMPTOMS: GENERAL

- [x] None
- [ ] Heaviness in limbs, back or head. Backaches, headache, muscle aches. Loss of energy and fatigability
- [ ] Any clear-cut symptom

14. GENITAL SYMPTOMS
(loss of libido, menstrual disturbances)

- [x] Absent

○ Mild

○ Severe

15. HYPOCHONDRIASIS

◉ Not present

○ Self-absorption (bodily)

○ Preoccupation with health

○ Frequent complaints, requests for help, etc. ...

○ Hypochondriacal delusions

16. LOSS OF WEIGHT

◉ No weight loss

○ Probable weight loss associated with present illness (>500g/week)

○ Definite weight loss(>1kg/week)

17. INSIGHT

◉ Not depressed (based on above items) OR Acknowledges being depressed and ill

○ Acknowledges illness but attributes cause to bad food, climate, overwork, virus, need for rest, etc.

○ Denies being ill at all

Supplementary S3. Assessment of FOG with a questionnaire

Question 1:“Do you feel that your feet get glued to the floor while walking, making a turn, or when trying to initiate walking ?”.

Answer:No

Yes

Question 2(if the answer of Question 1 is yes): Can you recall the specific time?

Answer:
